# Molecular determinants of prostate cancer metastasis

**DOI:** 10.18632/oncotarget.21085

**Published:** 2017-09-19

**Authors:** Kiera Rycaj, Dean G. Tang

**Affiliations:** ^1^ Department of Pharmacology and Therapeutics, Roswell Park Cancer Institute, Buffalo, NY 14263, USA; ^2^ Cancer Stem Cell Institute, Research Center for Translational Medicine, East Hospital, Tongji University School of Medicine, Shanghai 200120, China

**Keywords:** metastasis, prostate cancer, cancer stem cells, molecular mechanisms

## Abstract

Metastatic cancer remains largely incurable and fatal. The general course of cancer, from the initiation of primary tumor formation and progression to metastasis, is a multistep process wherein tumor cells at each step must display specific phenotypic features. Distinctive capabilities required for primary tumor initiation and growth form the foundation, and sometimes may remain critical, for subsequent metastases. These phenotypic features must remain easily malleable during the acquisition of additional capabilities unique and essential to the metastatic process such as dissemination to distant tissues wherein tumor cells interact with foreign microenvironments. Thus, the metastatic phenotype is a culmination of multiple genetic and epigenetic alterations and subsequent selection for favorable traits under the pressure of ever-changing tumor microenvironments. Although our understanding of the molecular programs that drive cancer metastasis are incomplete, increasing evidence suggests that successful metastatic colonization relies on the dissemination of cancer stem cells (CSCs) with tumor-regenerating capacity and adaptive programs for survival in distant organs. In the past 2-3 years, a myriad of novel molecular regulators and determinants of prostate cancer metastasis have been reported, and in this Perspective, we comprehensively review this body of literature and summarize recent findings regarding cell autonomous molecular mechanisms critical for prostate cancer metastasis.

## INTRODUCTION

Metastasis is a multi-step process wherein cancer cells disseminate from the primary tumor, arrest in a distant tissue, and subsequently form a clinically detectable tumor mass. Metastases are derived from molecularly heterogeneous tumor cells, which undergo Darwinian selection accumulating oncogenic mutant alleles affording unique metastatic characteristics with the greatest advantages. Mutations affecting cell proliferation, survival, and stemness pathways are critical for tumor initiation. Metastases continue to depend on these genetic aberrations acquired during tumorigenesis, but require additional traits such as invasion and migration, and immune system evasion in order to successfully metastasize [1]. Large-scale genome sequencing of human tumors has provided little evidence for recurrent and specific metastasis-restricted mutations suggesting that acquisition of metastatic propensities is not directly conferred by ‘metastasis genes’ [2]. Indeed, evidence supports the notion of mutations giving rise to pleiotropic epigenetic alterations in the acquisition of a metastatic phenotype. In addition, stress from targeted therapy leads to additional survival and growth mechanisms found in drug-resistant cancer-cell clones, as well as supportive interactions with the tumor microenvironment, driving rapid relapse and therapeutic resistance.

**Figure 1:**
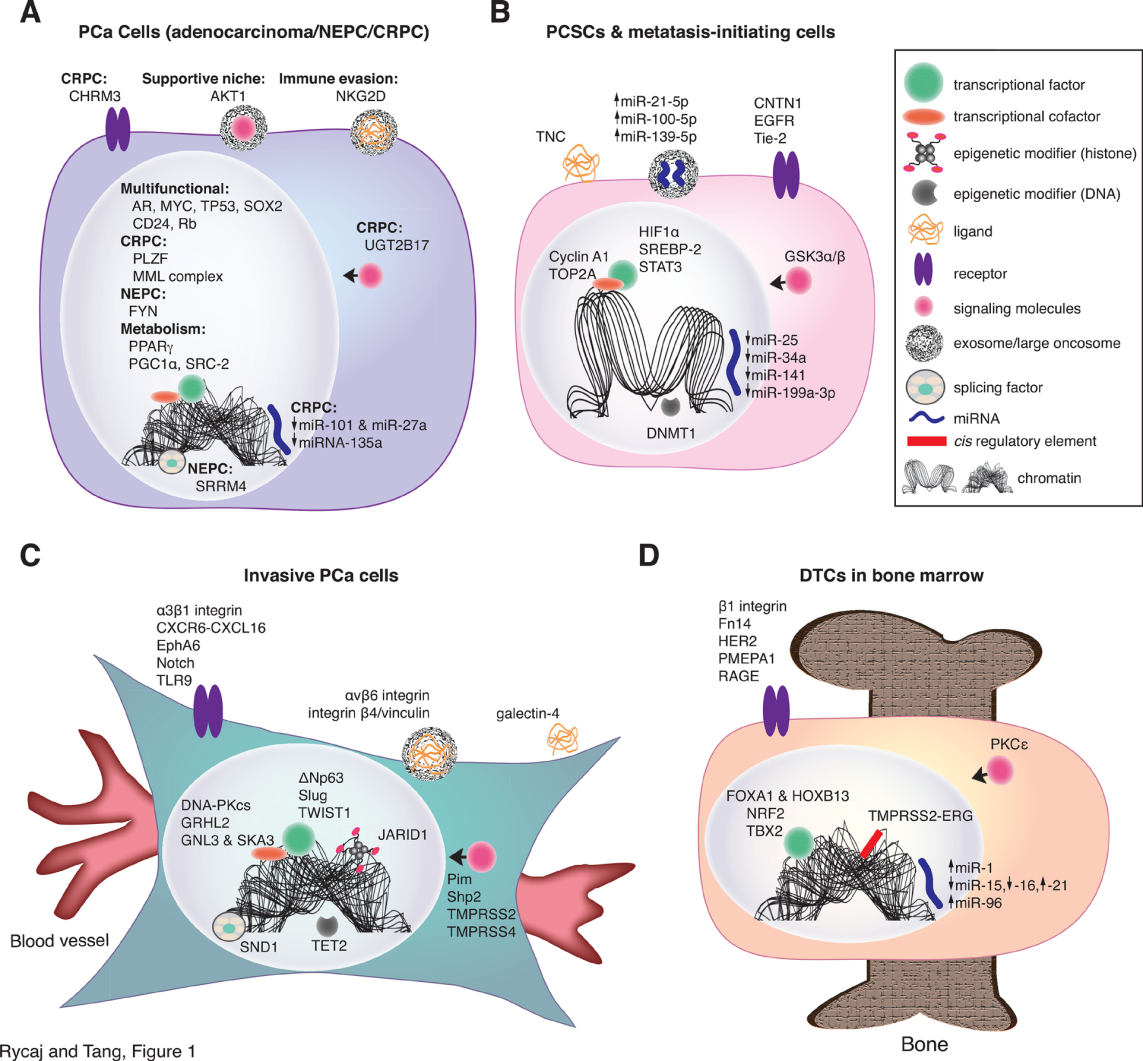
Novel molecular determinants confer specific phenotypes in PCa cells conducive to metastasis (**A**) *Novel molecular determinants in bulk (general) PCa cells discussed in this Perspective.* The majority of PCa is diagnosed as adenocarcinomas with most cells being positive for AR and differentiation marker PSA. A small percentage (~1%) of tumors are diagnosed as AR-negative undifferentiated NEPC. A significant portion (20-25%) of CRPC present NEPC phenotype expressing some neuroendocrine markers such as chromogranin A and synaptophysin. Shown is an image representing these 3 different PCa cell phenotypes and the novel cell-autonomous molecular determinants that contribute to metastatic capabilities in these cells. Illustrated here are representative molecular determinants that endow PCa cells a variety of capabilities including survival in androgen-deprivation conditions, establishing supportive niches, evading the immune system, and metabolic reprogramming. Multifunctional TFs and cofactors include those that confer metastatic capabilities in PCa cells. miRNAs altered in PCa cells can play tumor-suppressive 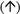 or oncogenic (onco-miR; 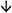) functions. Icons and their corresponding labels are shown in top right. See detailed discussions in the Text. (**B**) *Molecular determinants in PCSCs and metastasis-initiating cells.* PCSCs adopt many strategies in order to survive and initiate metastasis in foreign environments. For example, PCSCs express TNC, which functions in a non-canonical manner to aid in overcoming immune surveillance. CSCs transition between interchangeable states, regulated by the microenvironment, and this phenotypic plasticity (i.e., EMT, metabolic, tumor-initiating capacity) can play an important role in metastasis. Differentiation programming is dependent on unique combinations of *cis* and *trans* regulatory molecules, which cooperatively influence chromatin structure. Thus, chromatin structures differ between PCSCs and their differentiated progeny, as indicated by an “open chromatin” conformation in PCSC depicted. Tumor-suppressive miRNAs including miR-25, miR-34a, miR-141, and miR-199a-3p are generally devoid in PCSCs whereas onco-miRs such as miR-21 may be secreted by PCSCs in exosomes. Evidence suggests that CSCs are generally smaller than their differentiated progeny (compare with the cell in A). (**C**) *Molecular determinants in invasive PCa cells.* PCa cells become phenotypically more fibroblastic during the acquisition of invasive and migratory abilities, facilitating their intravasation into the blood vessels. Representative novel molecular determinants discussed in the Text are illustrated here. (**D**) *Molecular determinants in DTCs in the bone marrow.* Disseminating PCa cells may rely on unique molecules to home to more permissible distant microenvironment such as the bone. An example of dysregulation of a protein-coding gene is exemplified by TMPRSS2-ERG, which increases bone tropism of PCa cells. Once in the bone, DTCs can exploit various pathways in order to survive and initiate colonization.

Metastasis is the predominant cause of mortality from prostate cancer (PCa). In advanced PCa patients, initial treatment includes prostatectomy, followed by first-line hormonal therapy using GnRH analogs in an attempt to inhibit androgen receptor (AR) mediated signaling pathways. After a short span of regression, suppression of AR activity inevitably leads to an incurable recurrent disease state called castration resistant prostate cancer (CRPC), at which point the patient is put on second-line castration regimens to further suppress AR function (i.e., enzalutamide) and/or adrenal androgen biosynthesis (i.e., abiraterone). Despite these efforts, most patients ultimately develop resistance to these agents and the vast majority of CRPC patients develop metastases. As a potential mechanism of adaptive resistance to AR targeted therapy, a subset of patients with advanced CRPC may eventually evolve into an AR-independent phenotype, histologically displaying strong neuroendocrine (NE) characteristics. These NEPCs (neuroendocrine prostate cancers) display high metastatic propensities and are the most deadly and aggressive subset of PCa. Elucidation of the series of molecular alterations and various types of PCa cells that drive these and other fatal forms of PCa (Figure 1) will aid in the discovery and development of novel, efficacious therapeutics for both primary tumors and metastases. In this update, we summarize recent findings regarding cell autonomous molecular mechanisms critical for PCa metastasis.

## THE INVASION-METASTASIS CASCADE

Despite the lack of comprehensive biological mechanisms applicable to all metastatic diseases, progress has been made in uncovering unique molecular mechanisms involved in several steps of the metastatic cascade. During the invasion-metastasis cascade, cancer cells leave the primary tumor site, infiltrate surrounding tissue, enter the cardiovascular and/or lymphatic circulatory systems, and colonize distant organs. Throughout this cascade, the biological processes enabling metastases are supported by specific metabolic activities, highlighting the importance of metabolic reprogramming (reviewed in [3]). During dissemination, tumor cells acquire critical properties such as increased motility, invasiveness, and the ability to degrade components of the ECM (extracellular matrix), which allow them to exit the primary tumor site (Figure 1C). The process of epithelial-mesenchymal transition (EMT) can support many of the steps essential for tumor cell dissemination and distant metastasis. EMT in carcinoma cells is regulated by a series of EMT-associated transcription factor (EMT-TFs), which promote the loss of adhesive characteristics (epithelial traits) and the acquisition of invasive and migratory properties (mesenchymal traits) as well as the ability to overcome senescence, apoptosis, and anoikis [4, 5]. While once perceived as a distinct switch between two phenotypes, recent evidence supports a more poised, ‘partial EMT’ state, wherein cells retain both mesenchymal and epithelial traits, possibly a cellular state conducive to more efficient dissemination. After intravasation into the blood and/or lymphatic vessels, circulating tumor cells (CTCs) rely heavily on autonomous functions to increase migratory capacity and ensure survival in the circulation such as invadopodia formation, secretion of paracrine factors, proteases, and recruitment of stromal components and immunosuppressive cells. Once arrested in the capillaries of distant tissues, CTCs extravasate through endothelial walls into the parenchyma. Successful extravasation is dependent on interactions of disseminating tumor cells with unique microenvironments in distant organs and, in order to prime metastatic tumor cells for these microenvironments, the primary tumor stroma can select for organ-specific seeding traits. For example, homing strategies guide PCa cells to more permissible environments, such as the pelvic lymph nodes (LNs) and bone, in metastatic PCa (mPCa). Once extravasated into the parenchyma, disseminated tumor cells (DTCs) must adapt to and survive in foreign tissue microenvironments, a process called metastatic colonization. Accumulating evidence supports the notion that successful metastatic colonization depends critically on the ability of DTCs to evade immune surveillance, re-initiate tumor growth (i.e., to exit dormancy and re-start cell proliferation) and to employ organ-specific adaptive programs conferring survival advantages (Figure 1D) [4].

### Cancer stem cells (CSCs)

Tumor-initiating (or tumor-regenerating) capability is a cardinal characteristic of CSCs. Recent studies also indicate that CSCs may be endowed with a unique ability to metastasize (recently reviewed in [6]; Figure 1B). For example, CSCs have been shown to be critical for the formation and maintenance of metastasis in colorectal cancer (CRC) [7, 8]. Interestingly, the plasticity between CSCs and non-CSCs, and thus their functional contribution, is regulated by the surrounding niche environments [7, 8]. These studies support the idea that *stemness is a function that can be acquired at any time during the metastatic cascade*. The cellular plasticity may be based on distinct epigenetic states between CSCs and non-CSCs, and chromatin dynamics and lineage commitment are governed by signals from the stem cell (SC) niche. A recent study revealed distinct chromatin accessibilities of squamous cell carcinoma SCs (SCC-SCs) compared to normal counterparts (i.e., epidermal skin cells), indicating that large-scale chromatin remodeling had occurred during tumorigenesis [9]. During tumorigenic process, SCs responded to stress signals in the microenvironment by activating stress- and lineage-responsive transcription factors (TFs), which overrode normal regulatory elements and led to lineage plasticity [9]. Interestingly, the distinct open chromatin landscape was a combination of active signature genes from two distinct lineages of SCs capable of giving rise to SCC [9]. This blending of chromatin landscapes might also occur in PCa, considering that both basal cell layer SCs and luminal layer multipotent progenitor cells can function as a cell-of-origin for PCa (discussed in [10]). Additionally, our recent studies demonstrate that the gene expression profiles of basal/stem cells and luminal progenitors resemble those in advanced, aggressive, and castration-resistant PCa [10, 11]. Future assessment of chromatin landscapes in prostate cancer stem cells (PCSCs) may determine whether active signature genes are combined from the two distinct cell lineages.

The phenotypic plasticity associated with CSCs may overlap with the EMT spectrum, contributing to multiple stages of the metastatic cascade. Recent data provides evidence that mesenchymal and epithelial states in PCa cells contribute differentially in their capacities for tumor initiation and metastatic seeding, respectively [12]. Partial-EMT and mesenchymal-like tumor cells displayed enhanced stemness and invasiveness and enriched tumor-initiating capacity while only epithelial and MET tumor cells could form macrometastases due to their proliferative properties. Regardless if coupled or uncoupled with EMT, metastatic colonization is contingent upon the dissemination of CSCs to re-initiate tumor growth.

## ACQUISITION OF METASTATIC TRAITS

Cancer and, ultimately, metastasis, involves genetic alterations that lead to changes in the gene expression program, thus altering protein output, both quantitatively and qualitatively (Figure 1) [13]. This can occur when genetic alterations cause impairments in gene regulatory mechanisms at transcriptional, post-transcriptional or post-translational levels. The most efficient way to regulate gene expression is at the transcriptional level and many cancer-associated genes encode TFs and coregulators. TFs regulate gene expression by interacting with cofactors, recruiting the transcriptional machinery, including RNA polymerase II, and by binding to CREs (*cis*-regulatory elements) within gene regulatory regions in a sequence-dependent manner to initiate RNA synthesis. Enhancers control the transcriptional activity of promoters, and are frequently associated with TFs that define cell-type identity, and generate cell type-specific transcriptional responses (reviewed in [14]). In addition, alterations of components comprising intracellular signal transduction cascades such as ligands, cell surface receptors, and signaling molecules, are also prevalent in mPCa. During the acquisition of metastatic traits, integrated transcriptional networks evolve and are reprogrammed, and crosstalk between networks is extensive. Thus, oncogenic events can affect multiple metastatic capabilities.

Cancer is caused by an accumulation of genetic alterations conferring unique properties to cancer cells, including metastatic ability. Mutations are generated via several mechanisms including nucleotide substitutions, copy-number variations (i.e., insertions, deletions, duplications) and DNA rearrangements (i.e., inversions, translocations, chromothripsis, chromoplexy) [15]. Point mutations are relatively rare in PCa [16–18]. Instead, PCa involves large-scale genomic rearrangements and extensive copy number alterations, which often lead to loss of one or both copies of critical tumor suppressor genes as well as oncogenic fusions [19]. The prevalence of large-scale genomic alterations in mPCa is logical as acquisition of a metastasis phenotype includes multiple capabilities. Any factor that increases the mutation rate will increase the likelihood of cancer and its progression to metastasis. For example, epigenomic changes, DNA repair, and inflammation can dramatically enhance the acquisition of metastatic capabilities.

### Epigenomic changes

Epigenetic mechanisms control the transcriptional availability of the genome by directly modifying DNA, as well as altering chromatin structure. Mutations to the molecules that comprise and modify the chromatin landscape commonly underlie the altered gene expression profiles in metastatic carcinoma cells. Chromatin composition is dynamic and genetic and epigenetic mechanisms work cooperatively to enable the acquisition of capabilities necessary for cancer (reviewed in [20]). Epigenetic modifications, such as aberrant DNA methylation patterns and histone modifications, can alter the packaging of DNA, and thus DNA accessibility to TFs and the transcriptional machinery, inactivating tumor suppressor genes. Alternatively, TF affinity for a DNA motif can be modulated by interactions with chromatin-modifying enzymes or RNA cofactors, post-translational modifications of the TF, and by physical properties of the DNA fiber and surrounding chromatin. Once bound to DNA, these TFs can also change chromatin features such as histone modifications and DNA demethylation. Signaling kinases can also alter the chromatin structure directly. For example, protein kinases and other nuclear kinases can associate with chromatin in the nucleus and phosphorylate histone proteins.

### DNA damage repair

Acquisition of a metastatic phenotype can be aided by deregulated DNA damage repair mechanisms. Inactivation of genes encoding components of the DNA-maintenance machinery, via inactivating mutations or via epigenetic repression, is an efficient means of increasing the mutation rate and acquiring mutant genes crucial for metastasis (reviewed in [21]). In PCa, components of DNA damage response pathways have been implicated in the generation of gene fusions through chromosomal rearrangements and the enhancement of AR activity by functioning as co-regulators [22]. Mutations also arise as a consequence of genotoxic stress from cellular processes such as transcription that promote DNA damage and genomic rearrangements or exhaust DNA repair mechanisms. Scenarios such as oncogene activation, hormone signaling, and inflammation, are causes of these situations. For example, activation of the AR axis can contribute to double strand break formation. Mutations themselves can cause more mutations, as high rates of mutation cause genome instability, which can further generate random mutations that are advantageous for metastasis, further expediting their acquisition.

Next-generation sequencing (NGS) has revealed germline and/or somatic DNA repair deficiencies in PCa. In a cohort of lethal PCa, data suggested that DNA-repair defects might act as drivers of metastatic subclonal expansion [23]. Microsatellite instability and mutations in *BRCA2* were also observed as metastasis-exclusive in two cases. Increases in chromosomal instability were observed in parallel to metastatic progression in PCa [24]. Comparative analysis of data from several studies found that mPCa samples showed more frequent alterations in genes implicated in DNA repair [24–26]. In another cohort, men with advanced high-risk PCa were more than five times as likely to harbor either heritable or somatic mutations in DNA repair genes (i.e., *BRCA2*, *ATM, MLH1* and *MLH2*) than patients with low-risk tumors [27].

### Tumor-promoting inflammation & immune evasion

Acquisition of metastatic capabilities can also be fostered by inflammation, and several cell-intrinsic inflammatory mechanisms have been linked to metastatic progression in PCa (reviewed in [28, 29]). Cell intrinsic mechanisms include activation of various classes of oncogenes, which drive the expression of inflammation-related programs. In addition, the differential expression of chemokines/cytokines and their receptors by PCa cells can induce inflammatory responses that can facilitate the promotion of cell proliferation, invasion, metastasis-related tissue remodeling, subversion of adaptive immunity and a reduced response to hormones. DTCs residing in distant tissues must protect themselves from immune attack. DTC-intrinsic abilities such as suppressing T-cell proliferation and effector function, and inhibiting NK (natural killer) cell cytotoxicity, can contribute to a favorable immune suppressive microenvironment capable of promoting metastatic PCa colonization.

### Metabolic reprogramming

The transition between dormant and active (proliferative) states in DTCs during metastatic latency depends on functional adaptive programs, which confer survival advantages. For example, metabolic adaptations may support the energetic demands of tumor initiation and continuous cell growth and proliferation in distant metastatic sites. Indeed, recent data shows an increased glycolytic phenotype in advanced stages of PCa and a correlation with poor prognosis [30]. This and other data supports the concept that PCa cells make the metabolic switch to glycolysis only in the metastatic stage, and not early in the transformation process. There is also an observed CSC metabolic plasticity wherein cells exposed to a glucose-deprived and hypoxic tumor microenvironment (TME), shift to mitochondrial oxidative metabolism in order to support CSC properties [31]. Even CSC and EMT phenotypes require distinct metabolic dependencies. For example, major metabolic profiles were different between metastatic prostate epithelial CSCs and non-CSCs, and were independent of EMT [32]. Finally, PCa cells can exploit cells in the TME to generate metabolic intermediates that are in turn used by cancer cells. Metastatic PCa cells have the ability to modulate the metabolism of adipocytes via stimulating lipolysis in the effort of utilizing the fat cell-supplied lipids to fuel the glycolytic pathway [33].

### Other mechanisms that regulate PCa metastasis

Comparative studies of paired primary tumor and metastasis samples have allowed identification of genetic and epigenetic alterations that drive progression to metastatic disease. Comparative analysis of data from recent studies found the mutational and somatic copy number alteration burden to be significantly higher in PCa metastases than in primary PCa [24–26]. These results suggest substantial primary to metastasis genetic divergence in PCa. In terms of major oncogenic driver alterations between metastatic foci, data shows that substantial heterogeneity exists between men with mPCa, but limited diversity among metastases within an individual [34]. These sequencing efforts have provided evidence of alterations in genes encoding classical signaling proteins as well as novel genes involved in unique cellular processes such as epigenetic regulation, alternative splicing, and metabolism in mPCa.

It is important to keep in mind that it is unlikely that activation of a specific gene only depends on one TF or epigenetic molecule to modulate its transcription. TFs and epigenetic regulators can modulate a variety of downstream targets at the posttranslational level. Furthermore, intracellular signaling molecules regulated by TFs and epigenetic molecules can also cross talk with other regulatory pathways. Finally, the TME imposes profound effects upon cancer cells at distant sites, and interactions between metastatic cells and the TME govern metastatic seeding, survival, dormancy, colonization, and growth. Due to these complexities, this updated Perspective will be organized by the function of cell-intrinsic molecular determinants (Figure 1; Supplementary Table 1) and not by the major intermediate pathways they belong to or the ultimate metastatic capabilities they confer. The discussions offer new insights into the molecular mechanisms that determine metastatic progression in PCa.

## PROMETASTATIC ALTERATIONS: TRANSCRIPTIONAL CONTROL

Genetic alterations in cancer cells ultimately result in dysregulated transcriptional programs (reviewed in [13]). Transcriptional dysregulation can be due to genetic alterations in signaling factors converging on transcriptional control, or genetic alterations in gene control factors themselves (i.e., both TFs and chromatin modifiers).

### TFs

Several types of TFs deregulated in cancer cells include master TFs involved in conferring cell identity, TFs that amplify transcriptional output via controlling proliferation, and TFs involved in altering the control machinery in response to extracellular signals [13]. Master TFs establish and maintain transcriptional programs and the chromatin landscape that characterize cell type-specific differentiated states and thus survival of specific cancer cell subtypes. Master TFs can alter the enhancer function, which in turn modifies the binding landscape of oncogenic TFs. For example, the AR cistrome undergoes extensive epigenetic reprogramming in AR-driven bone metastasis [35]. Analysis showed that FOXA1 (forkhead box A1), a general pioneer factor, and HOXB13 (homeobox B13), highly lineage-specific factor, co-localized and reprogramed the binding landscape for AR and thus the AR cistrome, suggesting they play key roles in AR-driven bone metastasis [35]. In another study, novel SOX2 (SRY (sex determining region Y)-box 2) target genes drove NE (neuroendocrine) progression and spread of PCa [36]. Mechanistically, SOX2 upregulated NE differentiation genes, neurotrophins/neurotrophin receptors, pluripotency and EMT-TFs, angiogenic and lymphangiogenic factors, and promoted PCa cell invasiveness and motility [36]. Aberrant expression of lineage-specific TFs can also confer advantages to cancer cells, such as acquired resistance to targeted cancer therapies, via plasticity. For example, increased expression of SOX2 as a result of functional loss of *Tp53* and *Rb1* promoted lineage plasticity, which in turn promoted a shift from AR-dependent luminal epithelial cells to AR-independent basal-like cells thus enabling anti-androgen resistance [37, 38]. Finally, increased TBX2 (T-box transcription factor 2) expression activated an invasive and metastatic behavior in PCa cells, promoting bone metastasis and growth in the bone microenvironment that was mediated by the canonical WNT (*WNT3A*) promoter and its downstream effectors, MMP9 (matrix metallopeptidase 9), MMP2, and IL-6 (interleukin-6) [39].

Cell proliferation TFs are among the most frequently mutated genes in cancer. The late acquisition of *TP53* missense mutations was recently linked to expansion of metastatic subclones in lethal PCa [23]. In another cohort, a genomic landscape analysis revealed that alterations in *TP53* and *AR* were greatly enriched in mCRPC (metastatic CRPC) relative to primary PCa [24]. Comparative analysis of data from recent studies found that metastatic PCa samples showed more frequent alterations in TP53 [24–26]. Data from a cohort of 10,945 tumors from patients with advanced cancer (57% primary tumors and 43% metastatic tumors), including 623 PCa patients, revealed that many genes originally identified as significant in TCGA (The Cancer Genome Atlas) studies, were even more frequently mutated in the new cohort [40]. For example, in PCa, the frequency of *TP53* mutations was > 4-fold greater in the new cohort, further supporting the clinical relevance of this somatic mutation. Genomic amplification or transcriptional dysregulation of MYC is frequently seen in mPCa and combined MYC activation and *Pten* loss driven by the *Hoxb13* regulatory locus may synergize to induce genomic instability and aggressive mPCa [41]. The development and use of a novel GEMM (genetically engineered mouse model) in *Pten/Trp53*-deficient PCa provided functional validation of Myc as a driver of local metastasis and critical for maintenance of metastasis [42]. The study [42] showed that deletion of *Pten* and *Trp53* triggered metastasis, and these lesions showed activation of Myc in the absence of Akt activation. Another study highlighted IL-6/Stat3 signaling as a casual factor for MYC-driven metastasis after loss of *Pten* and *p53* [43]. IL-6 drove an AKT-MYC switch through activation of the AKT-suppressing phosphatase PHLPP2 (PH domain and leucine rich repeat protein phosphatase 2), and then initiated a downstream program of STAT3-mediated MYC activation, which drove cell proliferation and disease progression [43]. Secreted IL-6 also activated adjacent stromal proliferation through STAT3/MYC. This data supports the idea that inflammation contributes to the progression of PCa, and that inflammatory chemokines affect cell motility and proliferation. Finally, N-Myc and myrAKT1 (myristoylated and constitutively active AKT1) drove the transformation of human prostate epithelial cells to NEPC [44]. N-Myc/myrAKT1 transformed tumor cells were highly aggressive and proficient in the multi-step process of metastatic dissemination, and exhibited many molecular attributes found in human NEPC [44].

Signaling TFs alter the transcriptional machinery in response to extracellular signals by binding to enhancers occupied by master TFs. The most important ligand-activated TF in the context of normal prostate homeostasis and PCa is AR. Comparative studies have revealed that (treated) mPCa samples harbor more frequent alterations in AR than primary tumors [24–26]. The high frequencies of AR pathway alterations support the notion that the vast majority of mCRPC remain dependent on AR signaling. As PCa progresses, many changes occur in genes and pathways that depend on or are regulated by AR. For example, androgen-induced AR signaling inhibited *SPARCL1* (secreted protein acidic and rich in cysteine-like 1) gene expression through chromatin remodeling and facilitated PCa progression [45]. Mechanistically, direct AR binding and HDAC (histone deacetylase)-mediated deacetylation at the *SPARCL1* locus functioned cooperatively to suppress *SPARCL1* expression, by forming a closed chromatin conformation and hindering transcription [45]. In the context of c-MYC oncogenic activation, SPARCL1, a matricellular protein, inhibited both biological and biophysical properties associated with cellular migration and invasion such as dynamics of cytoskeletal remodeling, focal adhesion assembly, cell stiffness, and cell traction forces [45]. Interestingly, androgen/AR signaling may negatively modulate inflammatory and immune responses. For example, decreased AR signaling in luminal epithelial cells due to genetic inactivation caused cell-autonomous upregulation of cytokines and chemokines and impaired epithelial tight junctions [46]. This impairment allowed cytokines and chemokines to leak into periglandular spaces, thereby promoting immune cell infiltration [46]. A recent study reported that the majority of CRPC bone metastases (80%) showed high AR activity, high metabolic activity, and, intriguingly, low MHC class I expression and low numbers of infiltrating immune cells whereas the non-AR driven subgroup (20%) showed low AR and metabolic activities, but high MHC class I expression and immune cell infiltration [47]. The study also revealed an association between low tumor HLA class I immunoreactivity at diagnosis and poor clinical outcome, as well as markedly lower HLA class I expression in PC bone metastases compared to primary tumors [47].

STAT3 transduces signals from growth factors (GFs) and cytokine receptors on the cell surface and regulates the expression of genes that control cell proliferation, survival, and immune responses. Genetic inactivation of *Stat3* or *IL-6* signaling in a *Pten*-deficient PCa mouse model accelerated cancer progression leading to metastasis [48]. The study identified p19(ARF), a tumor suppressor, as a novel direct Stat3 target, and showed that loss of IL-6/Stat3 signaling disrupted the ARF-Mdm2-p53 tumor suppressor axis bypassing senescence and accelerating malignant progression [48]. On the other hand, STAT3 inhibition with galiellalactone significantly reduced tumor growth and early metastatic dissemination of PCa [49]. Moreover, AR downregulation induced STAT3 activation and promoted a PCSC phenotype via increased IL-6 expression [50]. These observations highlight potential context-dependent effects of STAT3 signaling in PCa growth and metastasis. Similarly, dysregulation and overexpression of HIF1A (hypoxia-inducible factor 1-alpha), the master transcriptional regulator of cellular response to hypoxia, by either hypoxia or genetic alternations have been implicated in cell survival, invasion, angiogenesis, and energy metabolism. In hypoxic PCSC-like cells, HIF1α level and HIF target gene expression were elevated, and upregulation of AKT occurred through a mechanism involving an mTOR/S6K/IRS-1 feedback loop [51]. Mechanistically, deregulation of the PI3K/AKT/mTOR pathway through HIF1α was critical for PCSC quiescence and maintenance by attenuating CSC metabolism and growth via mTOR and promoting survival by AKT signaling through insulin receptor substrate 1 (IRS-1) [51]. Importantly, HIF1α upregulation enabled resistance in PCSCs to selective mTOR inhibitors [51], providing an explanation for the low success rate of these inhibitors in PCa clinical trials. Androgen-deprivation therapy (ADT) can activate oncogenic programs by influencing signaling TFs. For example, PLZF (promyelocytic leukemia zinc finger) is a canonical androgen-regulated putative tumor suppressor gene in PCa, and may be a mediator of ADT resistance in a subset of CRPC by activating pro-growth pathways involving MAPK signaling [52]. Indeed, newly identified genomic alterations in a cohort of 150 mCRPC patients included those in ZBTB16/PLZF [24].

SREBP-2 (sterol regulatory element-binding protein-2), a TF that controls cholesterol biosynthesis and homeostasis in normal cells, has been found to play a novel role in promoting PCSC properties and PCa metastasis via transcriptional activation of c-MYC [53]. Overexpression of SREBP-2 induced PCa cell proliferation, invasion and migration, increased the PCSC population, prostasphere-forming ability, and tumor-initiating capability [53]. The TF p63 isoforms containing transactivation domains can efficiently transactivate p53 responsive genes, and conversely, ΔN proteins, which lack the canonical transactivation domain, promote cancer cell survival and tumor progression. One such isoform, ΔNp63, controlled migration via transcriptional regulation of MTSS1 (metastasis suppressor 1), leading to increased formation of membrane protrusions in highly metastatic PCa cells [54]. In a *Pten*-null background, PPARγ (peroxisome proliferator-activated receptor gamma), encoding a ligand-activated TF, was also identified as a promoter of mPCa through activation of lipid signaling pathways mediated by FASN (fatty acid synthase), ACC (acetyl-CoA carboxylase), and ACLY (ATP citrate lyase) [55]. NR2F1 (COUP-TF1), an orphan nuclear receptor, is a critical node in dormancy induction and maintenance by integrating epigenetic programs of quiescence and survival in DTCs [56]. NR2F1 was epigenetically upregulated (DNA promoter demethylation) in DTCs from PCa patients carrying dormant disease. Further, these PCa DTCs displayed a significant upregulation of a dormancy signature, wherein NR2F1-induced quiescence drove growth arrest via SOX9, RARβ and CDK inhibitors [56]. These newly emerged data support the notion that epigenetic reprogramming plays a role in dormant DTCs as they interconvert between dormancy and proliferation to establish metastases.

Master EMT-TFs, which are largely influenced by contextual signals, coordinate complex programs that control properties critical to PCa cell invasion and metastatic dissemination. Slug is overexpressed in cell clusters forming the invasion front of high-grade PCa, NED (neuroendocrine differentiation) areas, and lymph node metastasis, but down-regulated in most epithelial cancer areas [57]. Slug expression endowed PCa cells with highly malignant properties by regulating their self-renewal, and NED and metastatic potentials [57]. In clinical CRPC bone metastases, nuclear Twist, Slug, and Zeb1 localization and an EMT-like phenotype were shown to be present only in a small subset of cells [58]. Finally, a TWIST1-HOXA9 embryonic prostate developmental program seems to be ‘reactivated’ during PCa metastasis [59]. TWIST1 facilitated PCa metastasis by regulating *HOXA9* (homeobox A9) expression via cooperating with WDR5 (WD repeat-containing protein 5), a scaffolding protein, and lncRNA Hottip/HOTTIP, a long noncoding RNA, to increase H3K4 trimethylation at *HOXA9* promoter leading to invasion [59].

### Transcriptional cofactors

Transcriptional cofactors are recruited to enhancer-promoter regions by TFs to reinforce gene activation or repression. Alterations to the expression and activity of AR co-regulators in PCa are an important mechanism driving disease progression and therapy resistance. Recent evidence suggests that DNA-PKcs, the catalytic subunit of DNA-PK (DNA-dependent protein kinase complex) with an important role in DNA repair, is a clinically actionable driver of mCRPC by interacting with AR as a coactivator, to facilitate transcriptional activation of AR genes [60]. Additionally, DNA-PKcs negatively regulated the expression of UGT enzymes known to affect DHT (dihydrotestosterone) metabolism in CRPC, thereby implicating its role in therapeutic relapse. DNA-PKcs promoted pro-metastatic Rho/Rac signaling, resulting in DNA-PKcs-induced tumor cell migration and invasion. This DNA-PKcs activation was independent of DNA damage indicators, highlighting its non-canonical role in mPCa [60]. In some tumors, transcriptional cofactors become fused to chromatin regulators, producing gene-specific events. MLL (mixed-lineage leukemia) is a component of a large SET1-like HMT (histone methyltransferase) complex that possesses inherent H3K4 (histone 3 lysine 4) methyl transferase activity. The MML complex was recently identified as a crucial co-activator of AR in CRPC [61]. AR associated with the MLL complex via direct interaction with menin, an interaction that is required for AR-mediated gene expression, suggesting menin as a key mediator of aggressive PCa. In a non-canonical role, TOP2A (DNA topoisomerase 2 alpha) promoted aggressiveness by inducing chromosomal rearrangements of genes that contribute to a more invasive phenotype in PCa cells [62]. Mechanistically, TOP2A cooperated with AR to facilitate transcription of androgen responsive genes to promote tumor cell growth [62]. In another study, TOP2A was the most highly upregulated gene in recurrent and metastatic PCa [63]. Interestingly, TOP2A^neg^ and TOP2A^high^ PCa cells exhibited distinct molecular and tumor-propagating properties, with TOP2A^high^ representing the phenotype of recurrence/metastasis in PCa and a marker of rapid proliferation. TOP2A^high^ cells had the ability to initiate metastasis and showed more frequent abnormal cell divisions, while TOP2A^neg^ cell populations were enriched in CSCs [63]. These results support the notion that not all tumor-initiating cells posses the ability to metastasize. GRHL2 (grainyhead-like 2) was recently identified as an AR co-regulator that functioned as an enhancer of the oncogenic AR signaling pathway but also a suppressor of metastasis-related phenotypes [64]. GRHL2 maintained AR expression in multiple PCa model systems, was required for cell proliferation, enhanced AR's transcriptional activity, and co-localized with AR at specific sites on chromatin to regulate genes relevant to disease progression. These oncogenic functions were counterbalanced by its ability to suppress EMT and cell invasion, and evidence suggested that AR assisted GRHL2 in maintaining the epithelial phenotype [64].

CDKs (cyclin-dependent kinases) are also transcriptional co-activators and their dysregulation has been intimately associated with PCSC activities and with PCa progression and metastasis. For example, cyclin A1 regulated aromatase-associated pathways to promote metastatic homing and growth of PCSCs in the bone marrow [65]. Specifically, ALDH^high^ PCSCs facilitated metastatic growth by utilizing cyclin A1 and aromatase to increase androgen to estrogen conversion, and by recruiting MMP9 from the host bone marrow [65]. AMPA (aminomethylphosphonic acid), a glycine analog, inhibited PCa growth and metastasis by altering the expression of apoptotic and cell cycle regulatory genes [66]. AMPA inhibited PCa cell proliferation and metastasis by suppressing cyclin D1 expression, decreased BIRC2 (Baculoviral IAP Repeat Containing 2) expression to activate caspase 3 leading to apoptosis, and reduced the density of microvessels in the tumors [66]. mPCa samples showed more frequent alterations in RB1 [24–26], a protein involved in not only cell-cycle regulation but also transcriptional regulation of mitotic checkpoint genes. Loss of RB1 function was shown to be a key regulator of metastasis in mCRPC [67]. RB loss altered cytoskeletal organization, induced EMT, increased migration, invasion, and PCa metastasis via the RB/E2F regulation of motility receptor RHAMM (receptor for hyaluronan acid–mediated motility, which stabilizes F-actin polymerization by controlling ROCK signaling) [67]. Loss of RB function can also deregulate the expression of hypoxia-mediated transcriptional programs that govern angiogenesis, metastasis and NED, leading to acquisition of a more invasive phenotype and expression of NED markers [68]. In a mouse model, loss of *Rb1* and *Pten* in PCa derepresses epigenetic reprogramming factors, Ezh2 and Sox2, enabling epigenetic reprogramming toward a stem–like and androgen-unresponsive state [37]. This lineage plasticity facilitated metastasis, NEPC transformation, and resistance [37]. Mechanistically, *Rb1* loss facilitated lineage plasticity and metastasis of prostate adenocarcinoma initiated by *Pten* mutation, and additional loss of *Trp53* led to antiandrogen resistance [37].

SRC-2 (steroid receptor coactivator 2), a critical mediator of energy homeostasis, was recently implicated as a prominent metabolic coordinator of PCa metastasis, by imparting metabolic advantages to tumor cells [69]. SRC-2 promoted lipogenesis and reprogramming of glutamine metabolism in PCa cells resulting in increased survival and metastasis. In aggressive mCRPC, SRC-2 transcriptionally regulated fatty acid biosynthetic genes primarily by coactivating SREBP-1 (sterol regulatory element–binding protein 1), independently of the AR [69]. FYN, a SRC family kinase and a transcriptional co-regulator, also promoted a NED phenotype and the invasion and metastasis of NEPC cells [70]. The transcriptional co-activator PGC1α (peroxisome proliferator-activated receptor gamma co-activator 1α) has been proposed as a master regulator of metabolism in mPCa [71]. PGC1α suppressed PCa progression and metastasis via activation of an ERRα (estrogen-related receptor alpha)-dependent transcriptional program to elicit a catabolic state. Data in GEMMs and patient datasets demonstrated that the down-regulation of PGC1α in PCa represents a causal event for metastatic dissemination [71].

A recent study provided a missing link between the functional significance of glycoprotein CD24 overexpression in PCa and functional inactivation of *p53* and *ARF* (p14 Alternative Reading Frame) [72]. By inhibiting ARF binding to NPM (nucleophosmin), intracellular CD24 caused ARF destabilization leading to an increase in MDM2 (mouse double minute 2 homolog), which subsequently decreased p53 and p21/CDKN1A, resulting in increased tumor growth [72]. Supporting its role in mPCa, both targeted mutation and shRNA silencing of *CD24* reduced the growth, progression and metastasis of PCa [72]. Finally, one group identified *GNL3*, *MAT1A*, *SKA3*, and *ZMYM5* as novel PCa metastasis susceptibility genes [73]. Functional studies demonstrated that both *GNL3* (guanine nucleotide-binding protein-like 3) and *SKA3* (spindle and kinetochore associated complex subunit 3) had the greatest impact on *in vitro* cell growth, migration, and invasion. Their precise functions in regulating mPCa remain unknown [73].

### Chromosomal fusions and chromatin modulators

In PCa, the chromosomal rearrangement underlying the *TMPRSS2*–*ERG* (transmembrane protease serine 2-ETS-related gene) fusion leads to the overexpression of ETS family members including ERG. In a cohort of mCRPC patients, aberrations of ETS genes were frequent [24]. One study shows that TMPRSS2-ERG increased bone tropism of PCa cells and metastasis development by modulating transcription of genes involved in cell migration/adhesion and mechanisms known to be associated with bone physiology [74].

mPCa samples also harbored more frequent alterations in the KMT2C (histone lysine N-methyltransferase 2C) and KMT2D genes [24–26]. JARID1D, a lysine-specific demethylase frequently deleted in mPCa, functioned as an anti-invasion factor to suppress PCa progression [75]. Mechanistically, JARID1D suppressed the invasion, but not proliferation or migration, of PCa cells by repressing the invasion-associated genes MMP1, MMP2, MMP3, MMP7, and Slug via demethylating trimethyl H3K4 at their promoters [75].

### DNA methylation

DNA methylation alterations have been observed in lethal mPCa. One study revealed marked heterogeneity in DNA methylation profiles between men with lethal mPCa; however, each individual's distinct DNA methylation signature was tightly maintained across all disseminated metastases [76]. Importantly, epigenetic alterations in DNA methylation were comparable to genetic copy number alterations, suggesting that both have similar potential in serving as driver events during metastatic dissemination [76]. Another study validated eight differentially methylated CpG islands including CpGs in five genes (ALKBH5, ATP11A, FHAD1, KLHL8, and PI15) involved in regulatory functions, response to hypoxia, protein-binding, developmental processes, and ion transport, and three intergenic regions, between PCa patients with metastasis and patients with non-recurrent tumors [77]. How these differential methylation profiles enhance metastatic progression is unknown. In some cases, DNA methylation can regulate EMT and the CSC phenotype. For example, reduced expression of DNMT1 (DNA methyltransferase 1) played an important role in the induction of EMT and the CSC phenotype in PCa cells, with enhanced tumorigenesis and metastasis [78].

TET2 (ten-eleven translocation 2), a member of the TET family methylcytosine dioxygenases that catalyze the conversion of 5-methylcytosine to 5-hydroxymethylcytosine (5hmC), has been shown to bind to AR and its loss leads to increased PCa cell proliferation and invasion [79]. TET2 physically interacted with both AR and AR-coactivators to modulate androgen-AR signaling via the synthesis of 5hmC, which influenced expression of many genes encoding enzymes related to 2-oxoglutarate and succinate metabolism [79]. This suggests that TET2 is an energy sensor that modifies androgen-AR signaling, and based on the metabolic state of the cell, both genetic and epigenetic processes may positively modulate AR signaling in mPCa.

## PROMETASTATIC ALTERATIONS: INTRACELLULAR SIGNALING NETWORKS

Mutations have been discovered in key genes that encode members or regulators of signaling pathways, resulting in a metastatic phenotype. Indeed, aberrations of PTEN were frequent in a cohort of mCRPC patients, and new genomic alterations included those in genes also important in intracellular signaling such as PIK3CA/B, R-spondin, BRAF/RAF1, APC, and β-catenin [24].

### Signaling ligands

Cancer cells can produce GF ligands themselves resulting in autocrine proliferative stimulation, which can also stimulate normal cells within the tumor-associated stroma. PCa cells can produce pro-inflammatory cytokines and chemokines that attract TAM (tumor-associated macrophages) from the circulation, which then become tumor-specific and produce various GFs and cytokines and subsequently increase the survival and metastatic capabilities of PCa cells. Alternatively, PCa cells can also suppress immune cell functions. PCSCs from mPIN (mouse prostate intraepithelial neoplasia, a precursor lesion to murine PCa) used TNC (Tenascin-C), an ECM disulfide-linked hexameric glycoprotein, as a strategy to overcome immune surveillance [80]. TNC exerted its effects by inhibiting T-cell proliferation and effector functions via interacting with α5β1 integrin on the cell surface of T cells [80]. Additionally, PCSCs from both prostate draining lymph nodes and mPIN lesions expressed CXCR4 (C-X-C chemokine receptor type 4), which was essential for PCSC-mediated tumor metastasis, and migrated in response to CXCL12 (chemokine stromal cell derived factor-1 (SDF-1)), which was overexpressed specifically in the lymph nodes upon mPIN development, suggesting a homing strategy induced in PCSCs [80]. PCa cells can also ‘deliver’ signaling ligands in exosomes to mediate immune suppression and tumor immune escape. Exosomes derived from CRPC patients expressed ligands on their surface to downregulate cell-surface activating receptor NKG2D (natural killer group 2D) on NK and CD8^+^ T cells, leading to impaired cytotoxic function [81]. Interestingly, a large cohort of mPCa patients overexpressed autoantibodies against the glycoprotein fetuin-A, suggesting its potential use as an early indicator of metastatic disease [82]. Based on the known functions of fetuin-A in inhibiting bone proteins and ectopic bone formation, the authors proposed that anti–fetuin-A antibodies may neutralize fetuin-A in the serum to promote bone deposition in mCRPC [82].

### Transmembrane proteins and receptors

Aberrant glycosylation of surface proteins expressed by tumor cells can regulate intracellular and intercellular signaling to promote invasiveness, dissemination, and metastasis. Lectins can play a part in this process by recognizing glycan proteins, and then altering cellular processes such as adhesion of tumors cells to the ECM or endothelium, thus favoring dissemination. For example, galectin-4 (β-galactoside–binding lectin-4) was recently identified as a driver gene governing PCa metastasis [83]. Galectin-4 activated receptor tyrosine kinases or RTKs (EGFR, HER2, HER3, IGF1R), pERK, pAkt, fibronectin and Twist1, and reduced expression of E-cadherin, thereby facilitating EMT, invasion, and metastasis [83]. Upregulation of C1GALT1 (core 1 synthase, glycoprotein-N-acetylgalactosamine 3-beta-balactosyltransferase, 1)-mediated *O*-glycosylation was required to mediate galectin-4 binding and RTK activation, suggesting that concomitant changes in *O*-glycosylation and galectin-4 overexpression in PCa cells induced EMT-like phenotypes and metastasis [83].

The invasive and migratory capabilities of cancer cells can be supported by inflammatory mechanisms. Chemokine and cytokine receptors are frequently altered in cancer, and can support the collective cytoskeletal rearrangement and cell polarization in tumor cells during collective invasion. For example, the CXCR6-CXCL16 axis was shown to mediate both cellular adhesion and motility via regulating Ezrin-Actin polymerization and αvβ3 integrin clustering at the leading edge of the invasive front in aggressive PCa [84]. This occurred in a FAK (Focal Adhesion Kinase)/PI3K/PKC-dependent manner, resulting in enhanced migration, invasion and adhesion to endothelial cells ultimately leading to PCa metastasis [84]. Fn14 (TNFRSF12A) is the receptor for TWEAK (TNF-related weak inducer of apoptosis), a cytokine produced by infiltrating immune cells, and was shown to promote PCa bone metastasis via the p50/p65 NFκB pathway [85]. Authors proposed that Fn14-expressing PCa cells produce autocrine TWEAK in addition to TWEAK secretion from infiltrating inflammatory cells [85], suggesting that both cell-autonomous and non-autonomous Fn14-mediated functions may contribute to PCa bone metastasis. Importantly, this TWEAK–Fn14 axis promoted local inflammation due to NF-κB induction of inflammatory proteins, including cytokines, chemokines, adhesion molecules, and metalloproteases [85] suggesting a mechanistic link between PCa bone metastasis and inflammation. The results also established an association between Fn14 and low AR activity [85], suggesting that Fn14 may be a survival factor in PCa cells with low AR activity. Finally, autocrine cholinergic signaling promoted PCa growth and castration resistance - endogenous acetylcholine activated CHRM3 (cholinergic muscarinic receptor 3) in PCa cells, promoted CaM/CaMKK–mediated phosphorylation of Akt, induced PCa cell migration by regulating EMT, and conferred castration resistance [86].

The growth factor receptor EGFR has been shown to accelerate PCa bone metastasis by downregulating miR-1, a tumor suppressor in PCa, and activating oncogenic TWIST1 [87]. EGFR may also promote survival of PCSCs and CTCs that have metastasized to the bone [88]. A significant association between RANK and HER2 protein (i.e., ERBB2) overexpression in PCa bone metastasis was also observed [88], suggesting that this signaling axis may be pertinent to mPCa. Strikingly, MET (hepatocyte growth factor receptor) was almost exclusively expressed in mCRPC and PCa bone metastasis and not in other locations such as primary PCa or LN metastasis [89]. EphA6 (ephrin type-A receptor 6), an RTK, was recently identified as a potential novel PCa metastasis gene [90]. Overexpression of EphA6 promoted vascular and neural invasion in mPCa, in part by regulating the gene expression of PIK3IPA, AKT1, and EIF5A2 (eukaryotic translation initiation factor 5A2) [90]. Tie-2 is an RTK activated by Ang-1 (angiopoietin-1). PCSCs maintained their stemness via the Ang-1/Tie-2 signaling pathway that functioned as an autocrine loop [91]. The Tie-2 expressing PCa cells were capable of metastasizing to the bone, resistant to Cabazitaxel, and more adhesive to both osteoblasts and endothelial cells [91], suggesting that Tie-2 may play an important role during the development of mPCa. The TLR (toll-like receptor) family plays a role in activation of innate immunity. TLR9 expression and activation can trigger signaling cascades that lead to proinflammatory cytokine responses. Increased expression of TLR9 was associated with a higher probability of LN metastasis in PCa, and exerted its function via regulation of a series of invasion and migration-related genes, including CXCR4, MMP2, MMP9, and IL-8 [92]. Notch signaling has a multifaceted role in PCa progression. In *Pten*-null mice, Notch signaling was not required for the initiation of PCa, but did promote EMT and FOXC2 (forkhead box protein C2)-dependent tumor metastases [93].

Integrins play a role in cell attachment to other cells and to the ECM, and in the transduction of signals, and are thus important in conferring invasive and migratory capabilities in metastatic cells. The development of cohesive PCa cell clusters, as well as alterations in the composition of the basement membrane and interactions between ECM proteins, involve the LBI (laminin-binding integrin family)-axis (reviewed in [94]). Amplification, mutation, and many other alterations in essential genes in the LBI-axis have been causally implicated in PCa metastatic progression [95]. Transferring integrins via exosomes can promote mPCa. αvβ6, an epithelial-specific integrin, was efficiently transferred via exosomes to αvβ6-negative recipient cells to promote their invasion and migration [96]. Exosomes from taxane-resistant PCa cells were found to contain more integrin β4 and vinculin [97]. Cellular adhesion through integrin receptors can promote PCa cell escape from dormancy by reactivating proliferation leading to metastasis [98]. PCa cell-cell contact on bone marrow stroma induced cell proliferation via activation of β1 integrin associated with downregulation of TGFB2 signaling and upregulation of MLCK (myosin light chain kinase) activation and CDK6 (cyclin dependent kinase 6) [98]. Integrins can also modulate AR expression and function. For example, expression of αvβ6 was sufficient to promote aggressive PCa growth and CRPC via JNK1-mediated activation of AR [99]. Specifically, activated JNK1 promoted AR nuclear shuttling, concomitant ligand-independent AR transcription and subsequent survivin-regulated tumor growth [99]. Integrins, such as α3β1, can also negatively regulate mPCa-α3β1 integrin suppressed PCa cell migration, invasion, and anchorage-independent growth via signaling through Abl kinases to restrain Rho GTPase activity, and thus supporting Hippo pathway suppressor functions [100].

Advanced PCa cells express RAGE (the receptor for advanced glycation endproducts), a transmembrane receptor of the immunoglobulin super family, on their cell surface, which bound PR3 (proteinase 3), a serine protease present in inflammatory neutrophils and hematopoietic cells within the bone marrow microenvironment, thus mediating homing of PCa cells to the bone marrow [101]. This RAGE-PR3 interaction induced PCa cell motility through a non-proteolytic signal transduction cascade involving activation and phosphorylation of ERK1/2 and JNK1. CNTN1 (contactin 1), a neural cell adhesion protein, was preferentially expressed in PCSCs and promoted tumor regeneration, invasion, progression and metastasis, via AKT activation and reduced E-cadherin [102]. In another study, TGF-β (transforming growth factor-β) increased the expression of genes in PCa cells that regulate multiple steps of the metastatic cascade to the bone. The most upregulated gene was PMEPA1 (prostate transmembrane protein androgen induced-1), which was part of a negative feedback loop and served as a TGF-β signaling regulator to suppress PCa metastases to bone, via interacting with SMAD2/3 and HECT E3 ubiquitin ligases [103]. Only membrane-anchored isoforms of PMEPA1 interacted with SMADs and ubiquitin ligases, blocking TGF-β signaling independently of the proteasome.

### Serine proteases

Deregulation of pericellular proteolysis is involved in cancer progression due to its role in the degradation of the ECM and the alteration of the microenvironment. TMPRSS2 (transmembrane protease serine 2), an androgen-regulated membrane-anchored serine protease, stimulated a proteolytic cascade that mediated androgen induced PCa cell invasion, tumor growth, and metastasis [104]. The substrate for TMPRSS2 was matriptase, a cell surface proteolytic enzyme, and increased matriptase activation was associated with enhanced degradation of ECM proteins nidogen-1 and laminin β1. On the other hand, TMPRSS4 may regulate PCa metastasis and cancer progression by inducing Slug and cyclin D1 expression through activation of transcription factor AP-1 (activator protein 1) [105].

### Protein kinases and phosphatases

As discussed above, many protein kinases, especially RTKs including ERBBs, MET, and Tie-2 can regulate numerous cell-signaling pathways important for survival, proliferation, invasion, migration, and angiogenesis during PCa progression. In an effort to comprehensively define critical kinase signaling pathways in lethal mCRPC patients, one group developed a tool called pCHIPS (phosphorylation-based cancer hallmarks using integrated personalized signatures) [106]. Several novel signaling proteins were identified, including PRKDC (DNA-dependent protein kinase, catalytic subunit, DNA-PKcs), PRKAA2 (5′-AMP-activated protein kinase catalytic subunit alpha-2), PTK2 (protein tyrosine kinase 2), and RPS6KA4 (ribosomal protein S6 kinase alpha-4), as possible therapeutic targets and/or biomarkers in PCa. Interestingly, the transcriptional regulators were more consistent across the metastatic samples whereas the kinase activities varied. The multi-omic approach based on phosphoproteomic, gene expression, and transcriptomic data led to phosphorylation-based cancer hallmarks including cell-cycle, DNA repair, AKT/mTOR/MAPK, the nuclear receptor, migration/invasion, and stemness pathways in mCRPC [106]. Malignancy can associate with overexpression of wild type kinases with functional activity, or expression of an oncogenic mutant. In PCa, mutationally activated kinases are rare, while the overexpression of wild-type kinases has linked nonmutated kinases and their pathways to progression, castration resistance, and metastasis [107]. A recent study identified five wild-type kinases that promoted PCa bone and visceral metastasis including all three RAF family members (i.e., A-, B-, and C-RAF), MERTK (receptor tyrosine-protein kinase Mer), and NTRK2 (neurotrophin tyrosine kinase 2) [108]. In contrast to the well-established metastasis-promoting ability of RAF family members, neither MERTK nor NTRK2 have been previously implicated in mPCa.

Upregulation of Shp2 (Src homology region 2-containing protein tyrosine phosphatase 2), a tyrosine phosphatase that acts to amplify signals emanating from RTKs or cytoplasmic tyrosine kinases, promoted PCa metastasis by attenuating the PAR3/PAR6/aPKC protein complex, resulting in disrupted cell polarity, dysregulated cell–cell junctions and increased EMT [109]. High expression of TOPK (T-LAK cell-originated protein kinase), a protein kinase that plays a positive regulatory role in proper chromosomal separation and cytokinesis, in PCa CTCs promoted metastasis [110]. TOPK expression was modulated through the PI3K/PTEN and ERK pathways and increased TOPK enhanced CTC migration and/or invasion [110]. TOPK, also called PBK (PDZ-binding kinase), induced an aggressive pro-metastatic gene expression program in PCa cells via β-catenin-TCF/LEF-mediated MMP-2 and -9 production, increasing their invasive ability [111]. Pim serine/threonine kinase, which is mainly involved in regulation of cell proliferation, survival as well as motility, was associated with enhanced angiogenesis and lymphangiogenesis in PCa [112]. Furthermore, Pim expression increased phosphorylation of CXCR4, which may enable PCa cells to migrate towards tissues that express the CXCL12 chemokine ligand. PKCε (protein kinase C epsilon), an isozyme in the serine-thereonine kinase family, was identified as an essential mediator of PCa bone metastasis [113]. PKCε influenced expression of IL-1β, a cytokine implicated in skeletal metastasis, and was required for transendothelial cell migration and for the growth of PCa cells in a bone environment. Isoforms of GSK3 (glycogen synthase kinase-3), serine/threonine protein kinases which play roles in various cellular processes, were found to display distinct molecular and cellular mechanisms in PCa growth and micrometastasis. The regulation of cell survival, proliferation, induction of CSC-like properties, and rate of tumor growth in both early and advanced PCa were predominantly dependent on GSK3α [114]. In contrast, the promotion of EMT and acquisition of invasive and metastatic properties was more dependent on GSK3β-mediated inhibition of β-catenin expression and destabilization of cell-cell contacts [114]. Extracellular vesicles can also serve as active signaling platforms. mPCa cell-derived large oncosomes (LO), atypical extracellular vesicles, promoted the establishment of a tumor-supportive environment by inducing reprogramming of fibroblasts in the stroma [115]. LOs harbored sustained AKT1 kinase activity, and internalization induced reprogramming of human normal prostate fibroblasts as reflected by high levels of α-SMA, IL-6, and MMP9. In turn, LO-reprogrammed prostate fibroblasts stimulated endothelial tube formation *in vitro* and promoted tumor growth in mice. Activation of stromal MYC was critical for this reprogramming and for the sustained cellular responses elicited by LO [115].

### Transferases

A recent study [116] identified a novel function for UGT2B17 (UDP-glucuronosyltransferase 2B17), a membrane-bound enzyme localized on the cytosolic side of the endoplasmic reticulum that normally maintains androgen homeostasis in the prostate. UGT2B17 stimulated PCa cell proliferation, invasion, and progression to CRPC after androgen deprivation [116]. Specifically, UGT2B17 suppressed androgen-dependent AR transcriptional activity but enhanced ligand-independent activation of AR signaling by kinase pathways via interacting with and activating c-Src kinase, which in turn stimulated AR phosphorylation and activation, enabling CRPC progression [116].

## PROMETASTATIC ALTERATIONS: CO & POST-TRANSCRIPTIONAL CONTROL

Gene expression can be co-transcriptionally regulated at the RNA level by alternative splicing (AS). Alterations in the AS process can contribute to cancer [117]. This can be attributed to mutations in splice-site sequences, genes encoding spliceosomal proteins, as well as mutations affecting the splicing of key cancer-associated genes. A post-transcriptional mechanism involves the interaction of small non-coding miRNA molecules (i.e., ~20–22 nt mRNAs that do not encode proteins) and target mRNA transcripts. miRNA-induced gene silencing occurs either through translational silencing of the mRNA or through degradation of the mRNA via complementary binding.

### RNA splicing

Transcriptome-wide remodeling of AS can regulate processes underlying metastatic colonization in PCa. For example, a recent study revealed a large network of AS events enriched for pathways important for cell signaling and motility, which affected key regulators of the invasive properties such as CD44 and GRHL1 (grainyhead-like transcription factor 1), a master transcriptome regulator [118]. In terms of dysregulated spliceosomal proteins, data identified a novel function for SND1 (Tudor-SN; p100), a ubiquitous protein mainly known as a transcriptional co-activator, as a regulator of AS that promoted PCa cell growth, survival and migration [119]. Mechanistically, SND1 interacted with SAM68 (SRC associated in mitosis of 68 kDa), an RNA binding protein, and spliceosomal components on CD44 pre-mRNA, to regulate AS of the variable exons of the CD44 transcript [119]. Inclusion of the variable exons in CD44 correlated with increased proliferation, motility and invasiveness of PCa cells. Another study [120] identified a NEPC-specific RNA splicing signature that was predominantly controlled by the RNA splicing factor SRRM4 (serine/arginine repetitive matrix 4). SRRM4 drove NEPC progression through AS of multiple genes including REST (RE1 silencing transcription factor), a master regulator of neurogenesis [120]. AR pathway inhibition enhanced SRRM4 stimulation in adenocarcinoma cells to express NEPC biomarkers. Additional data suggested that AR pathway inhibition, genomic abnormality and deregulated AS programs likely cooperated to drive NEPC progression.

### miRNAs

Numerous miRNAs have been implicated in regulating the growth, castration resistance, stemness and metastasis of PCa [121]. miRNAs may either promote (i.e., Onco-miRs) or suppress tumorigenic and metastatic processes. miR-194 stimulated migration, invasion, and EMT in PCa cells and enhanced metastasis, implicating this miRNA as a potential driver of PCa metastasis [122]. miRNA-194, which is under the control of GATA2, promoted PCa metastasis by inhibiting SOCS2 (suppressor of cytokine signaling 2), resulting in derepression of the oncogenic kinases FLT3 and JAK2 and enhanced ERK and STAT3 signaling [122]. Another study demonstrated oncogenic and metastatic properties of miR-96 [123]. TGFβ-Smad signaling regulated the level of miR-96, which promoted PCa bone metastasis by downregulating AKT1S1, an AKT substrate, and enhancing mTOR activity [123]. Mechanistically, miR-96 targeted the 3′-UTR (3′-untranslated region) of *AKT1S1* mRNA leading to its downregulation and disruption of the AKT1S1-mTOR complex. Exosomes released from PCa cells and PCSCs may also contain unique miRNAs that can modify the local or premetastatic niche, thus facilitating PCa progression and metastasis. For example, exosomes from PCSCs contained abundant miR-100-5p and miR-21-5p, which were highly correlated with PCa malignancy, fibroblast proliferation, differentiation and migration, and tumor angiogenesis, compared to bulk exosomes [124]. Additionally, miR-100-5p, miR-21-5p and miR-139-5p found in PCSC exosomes increased the expression of MMP-2, -9 and -13 and RANKL and fibroblast migration [124].

mPCa exhibits reduced levels of miR-101 and miR-27a, suggesting these two miRNAs may possess metastasis-suppressive functions [116]. In support, both miR-101 and miR-27a inhibited expression of COUP-TFII (COUP transcription factor 2), FOXM1 (forkhead box M1), and CENPF (centromere protein F), the latter two of which represent master regulators of metastasis in PCa [125]. Further studies also implicated COUP-TFII as a master regulator of the metastatic network in mPCa. Importantly, this miRNA-COUP-TFII-FOXM1-CENPF regulatory axis was also involved in the development of enzalutaminde resistance [125]. In another study, loss of miR-15 and miR-16, in conjunction with increased miR-21 expression, aberrantly activated TGF-β and Hedgehog signaling, leading to increased local invasion, distant bone marrow colonization and osteolysis by PCa cells [126]. miR-34a inhibits PCSCs and metastasis by directly repressing CD44 [127]. miR-34a also directly targets *TCF7*, a WNT signaling-related gene, resulting in an inhibition of bone metastasis and cell proliferation in Ras-activated PCa, and directly interfered with the expression of the anti-proliferative *BIRC5* [128]. This data suggests a novel regulatory mechanism of combinatorial Ras and WNT signaling in advanced PCa [128]. As a tumor suppressive miRNA, miR-25 modulated the invasiveness and dissemination of highly metastatic PCSCs by interacting with the 3′-UTRs of proinvasive αv and α6 integrins [129]. miR-199a-3p suppresses the expansion and tumorigenic capabilities of PCSCs via targeting CD44 and several mitogenic molecules including c-MYC, cyclin D1 and EGFR [130]. miR-141, a miR-200 family member, functions to suppress PCSCs and metastasis via targeting a cohort of pro-metastasis genes including the Rho GTPase family members (i.e., CDC42, CDC42EP3, RAC1 and ARPC5) and stem cell molecules CD44 and EZH2 [131]. Interestingly, the expression and functions of miRNAs can be influenced by androgen. For example, loss of androgen-regulated miR-1 activated SRC and promoted PCa bone metastasis, establishing a mechanistic link between low canonical AR output and SRC-promoted metastatic phenotypes [132]. The expression of some miRNAs is epigenetically regulated by histone modification and/or DNA methylation, which can contribute to the upregulation of AR protein expression in CRPC. For example, epigenetic silencing of miRNA-135a resulted in increased AR axis activity under androgen-deprivation conditions [133].

## CONCLUSIONS

Metastasis represents a largely stochastic and complicated process that is ‘orchestrated’ by a large array of molecular determinants and deregulated signaling pathways. Traits that confer the metastatic properties can be acquired via protein-coding changes as well as noncoding variants that affect gene regulation or function. Recent progress on metastasis studies have identified many novel signaling pathway components such as ligands, receptors, and proteases, as well as novel regulatory mechanisms involving TFs, cofactors, and epigenetic modifiers (Figure 1; Supplementary Table 1), which together regulate the gene expression output necessary for metastasis. Co/post-translational regulatory networks including alternative splicing and miRNAs further modulate critical processes in mPCa. These complex, aberrant cellular regulatory networks form the mechanistic basis for PCa metastasis. Importantly, many of these molecules are involved in the regulation of PCSCs, a population of cells responsible for metastatic colonization and therapeutic resistance. Elucidation of these critical molecular alterations will facilitate the future development of novel metastasis-interfering therapeutics.

## SUPPLEMENTARY TABLE




