# Supplementary material for: Molecular determinants of prostate cancer metastasis
Source: Oncotarget. 2017 Sep 19;8(50):88211–31. doi: 10.18632/oncotarget.21085 (PMC5675705; doi:10.18632/oncotarget.21085)
Supplement: Supplementary file 1 [file oncotarget-08-88211-s001.pdf]

Molecular determinants of prostate cancer metastasis

SUPPLEMENTARY MATERIALS

Supplementary Table 1: Genetic alterations in prostate cancer metastasis. See Supplementary_ Table _1
